# Development and Characterization of Polyamide-Supported Chitosan Nanocomposite Membranes for Hydrophilic Pervaporation

**DOI:** 10.3390/polym10080868

**Published:** 2018-08-04

**Authors:** Ewelina Chrzanowska, Magdalena Gierszewska, Joanna Kujawa, Aneta Raszkowska-Kaczor, Wojciech Kujawski

**Affiliations:** 1 Faculty of Chemistry, Nicolaus Copernicus University in Toruń, 7 Gagarina Street, 87-100 Toruń, Poland; ewelina@doktorant.umk.pl (E.C.); mgd@umk.pl (M.G.); joanna.kujawa@umk.pl (J.K.); 2 Institute for Engineering of Polymer Materials and Dyes, 55 Marii Skłodowskiej-Curie Street, 87-100 Toruń, Poland; aneta.raszkowska-kaczor@impib.pl

**Keywords:** composite membranes, chitosan nanocomposite, montmorillonite, Cloisite 30B, polyamide, surface properties, hydrophilic pervaporation, ethanol and isopropanol dehydration

## Abstract

An experimental protocol of preparation of homogeneous and nanocomposite chitosan (Ch) based membranes supported on polyamide-6 (PA6) films was developed and described in detail. Montmorillonite (MMT) and Cloisite 30B (C30B) nanoclays were used as nanofillers to improve mechanical properties of chitosan films. The surface, mechanical, and transport properties of PA6 supported Ch, Ch/MMT and Ch/C30B membranes were studied and compared with a pristine, non-supported chitosan membrane. Implementation of advanced analytical techniques e.g., SEM reveal the clays nanoparticles are well dispersed in the chitosan matrix. According to AFM images, composite chitosan/nanoclay membranes possess higher roughness compared with unfilled ones. On the other hand, an incorporation of clay particles insignificantly changed the mechanical and thermal properties of the membranes. It was also found that all membranes are hydrophilic and water is preferentially removed from EtOH/H_2_O and iPrOH/H_2_O mixtures by pervaporation. Supporting of chitosan and chitosan/nanoclay thin films onto PA6 porous substrate enhanced permeate flux and pervaporation separation index, in comparison to the pristine Ch membrane. Concerning separation factor (β), the highest value equal to 4500 has been found for a chitosan composite membrane containing Cloisite 30B contacting 85/15 wt % iPrOH/H_2_O mixture. The mentioned membrane was characterized by the normalized flux of 0.5 μm·kg·m^−2^·h^−1^. Based on the established data, it was possible to conclude that chitosan membranes are meaningful material in dehydration of azeotropic mixtures. Nevertheless, to boost up the membrane efficiency, the further modification process is required.

## 1. Introduction

Membrane separation techniques become very important and widely applied methods as an alternative to conventional separation processes, like extraction, distillation, and adsorption. Their main advantage is the fact that they are environmentally friendly, highly efficient and simultaneously characterized by low energy consumption [1]. Membrane separation techniques possess a wide spectrum of application in different areas e.g., food and beverage processing (separation, recovery of byproducts, purification) [2,3], hydrometallurgy (control of the pollution, metal ions recovery) [4,5], pulp and paper industry (recovery of chemicals, replacing evaporation process) [6] or chemical process industries (separation of organic solvents, gases, recovery of chemicals) [1,7,8]. The medical and pharmaceutical sector is also a broad consumer of different membrane techniques, including controlled release systems, blood fractionation or artificial kidney [9,10]. Moreover, the membrane technology is widely used in water and wastewaters treatment [11].

In recent decades, special effort has been devoted to developing membranes possessing improved separation and transport properties. The special attention was focused on biopolymer-based membranes exhibiting unique properties like biocompatibility, biodegradability, good chemical and thermal stability, non-toxicity and simplicity of chemical and physical modification [12,13,14]. What is even more important is that biopolymers are highly available and obtained from renewable sources, becoming therefore a good alternative to synthetic polymers derived from petroleum oil. 

The ability of biopolymers to form thin layers (e.g., by solution-casting and solvent evaporation) plays a crucial role in the preparation of such membranes. This feature is characteristic for different polysaccharides obtained from diverse sources, plants (e.g., starch), algae (e.g., alginates), or animal sources (e.g., chitosan—Ch). Chitosan is a derivative of chitin—the second most abundant organic compound in nature, after cellulose. Ch is produced mainly by chemical chitin deacetylation [15,16]. Structurally Ch is composed of randomly distributed mers of β-(1→4)-linked 2-acetamido-2-deoxy-β-D-glucopyranose and 2-amino-2-deoxy-β-D-glucopyranose [16]. Chitosan-based membranes have been tested in pervaporation (PV), ultrafiltration (UF), gas separation (GS), reverse osmosis (RO), controlled drug delivery and as an electrolyte and electrode material in polymeric electrolyte fuel cell [17,18,19,20].

Pervaporation is a membrane technique used for the separation of binary and multicomponent liquid mixtures. The applications of PV can be generally divided into three areas [21]: (i) hydrophilic pervaporation (dehydration of water-organic mixtures), (ii) hydrophobic pervaporation (removal of organic volatile compounds from aqueous solutions), and (iii) organophilic pervaporation (separation of mixtures of organic solvents). Because of the hydrophilic nature, chitosan membranes have been extensively tested in the dewatering of water/alcohol mixtures by pervaporation. It was found that pristine monolayer chitosan membranes possess the excellent separation properties, however permeate fluxes through these membranes are rather low, especially when temperature does not exceed 30 °C [22,23,24,25,26,27,28]. This drawback can be eliminated by decreasing the chitosan membrane thickness. The other disadvantage is related to the low resistance of chitosan to high water content solutions as well as its low mechanical performance [14,29]. Different methods have been proposed to improve chitosan membranes characteristics like bulk and surface crosslinking [17,29], nanocomposite formation [30], and casting of composite chitosan membranes on various porous supports [24].

Transport through dense pervaporative membranes is a solution-diffusion type in which diffusion through the membrane is the rate-determining step. Therefore, the presence of a filler can substantially affect this process. If a filler compatible with the chitosan matrix is used, it is located in the free volume within the polymer matrix, thus creating a tortuous path for the permeating molecules [31]. Qui et al. [32] found that the permeate flux in pervaporation of water/ethanol mixture increased significantly when functionalized multiwalled carbon nanotubes were added to the polymeric dope of chitosan. Dudek et al. [21] combined the crosslinking with the addition of iron oxide particles into the chitosan matrix, which resulted in the improvement of all separation parameters in H_2_O/EtOH dehydration. Hydrophilic zeolite-filled PVA and chitosan membranes prepared by Gao et al. [33] exhibited better separation factor and improved flux in the pervaporation separation of organic–water system. Although the extensive literature survey indicated that many chitosan-based natural mineral clay nanocomposite films were obtained, only Choudhari et al. [34] evaluated their application in PV process.

Tuning of the membrane structure, namely by preparation of composite chitosan-based films, allows not only to improve mechanical stability, but also to enhance transport features (i.e., higher permeate flux) in comparison to the homogeneous structures. The main advantage causing the abovementioned properties is a possibility to reduce the thickness of the selective layer deposited on the porous structure. The lower the mass transport resistance of porous support, the better the permeability of the composite membrane. Wang et al. [35] used hydrolyzed polyacrylonitrile (PAN), Huang et al. [25] and Ghazali et al. [28] applied a microporous polysulfone (PS) substrate. In addition, polyamide-6 (PA6) porous films were used for chitosan composite membranes fabrication [36,37,38], but until now such membranes were not applied in the PV process.

Albo et al. [39,40,41] drew attention to the fact that a clear understanding of polyamide membranes characteristics at macro- and nanoscale level is needed for the optimization of their separation performance. They have found that when PA6 is used as a separation layer deposited on porous support, different pretreatment procedures of membranes, i.e., different drying conditions (on air, in oven, by solvent exchange, by freeze-drying method), substantially influenced the average free-volume pore size of PA6 layer and thus their transport properties in gas separation tests and dehydration of isopropanol by pervaporation. These changes were attributed to the swelling/shrinkage of PA6 selective layer. Meier-Haack et al. [42] analyzed an effect of annealing temperature of asymmetric PA6 membranes on their selectivity in separation of water/isopropanol mixture by pervaporation. Based on the presented result it was concluded that drying of membranes above the glass transition temperature of polyamide-6 (~60 °C) caused the formation of crystalline domains which led to an increase in separation factor. In previous studies [43] we have also analyzed the influence of the composition of casting PA6 solution and drying temperature on the selectivity of resulting membranes in pervaporation process of water/ethanol mixtures founding the highest separation factor for membranes cast from 10 wt % PA6 solutions and dried at higher temperatures.

Based on the abovementioned findings, this research presents a fabrication method of novel polyamide-6 supported chitosan nanocomposite membranes, followed by the determination of separation and transport properties in dewatering of alcohol mixtures by pervaporation. Two different nanofillers were chosen for this research, i.e., neat montmorillonite and its modified derivative Cloisite 30B. An effect of hydrophilic nature of nanoclay on chitosan films surface properties was evaluated and compared with pristine chitosan film by scanning electron microscopy (SEM), atomic force microscopy (AFM), and contact angle measurements. The important part of this work was devoted to the comparison of mechanical properties of PA6 supported and non-supported chitosan films. Finally, the performances of membranes in water removal from ethanol and isopropanol binary aqueous solutions were examined and compared with pristine PA6 supported chitosan.

## 2. Materials and Methods

### 2.1. Materials

Commercially available chitosan from crab shells was purchased from BioLog Heppe GmbH (Landsberg, Germany). The degree of deacetylation (DDA) of chitosan determined by potentiometric titration was 72.25 ± 0.77% and the viscosity average molecular weight (*Mv*) of chitosan solutions was 148 ± 26 kDa.

**Figure 1:**
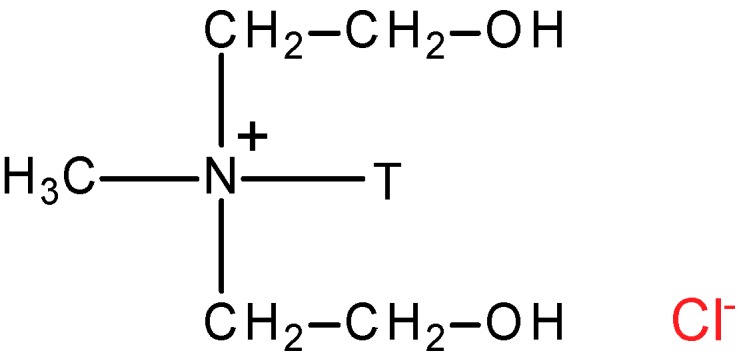
Structure of Cloisite 30B modifier (where T—tallow (~65% C18; ~30% C16; ~5% C14)).

Two different clay minerals were used in the presented study: montmorillonite (MMT) and its derivative Cloisite 30B (C30B). MMT was provided by the Riedel–de Haen and its chemical composition is given in Table 1. Main impurities of MMT (CaO, K_2_O, Fe_2_O_3_) do not exceed 4.8 wt % [44]. Cloisite 30B (C30B) is a MMT derivative modified with quaternary ammonium salt (MT2EtOH: methyl-tallow-bis-2-hydroxyethyl ammonium) (Figure 1).

Polyamide-6 (PA6) in a granulated form was provided by ZWCH STI-LON S.A. (Gorzów Wielkopolski, Poland). Formic and acetic acids, as well as dehydrated ethanol (pure 99.8%) and isopropanol (pure min. 99%) were purchased from Avantor Performance Materials Poland S.A. (Gliwice, Poland). Deionized water was used throughout the entire study.

### 2.2. Composite Membranes Preparation

Polyamide-6 support films were prepared using a non-solvent induced phase inversion method (NIP) according to previously reported procedure [45,46]. Briefly, PA6 was dissolved in the mixture of formic acid, acetic acid, calcium chloride and water (52.4:8.3:8.3:17.5 wt %) resulting in 13.5 wt % polymer solution. Subsequently, the PA6 solution was cast on a glass plate using a casting knife (0.4 mm slit), left on the air for 10 min (for an initial gelation) and then immersed into the non-solvent coagulation bath (water) at room temperature [45,46,47]. Precipitation occurred due to the exchange of solvent and non-solvent. The resulting porous PA6 membranes were dried for 24 h at room temperature.

Three different film-forming solutions were prepared, i.e., one composed of pure chitosan and two others were composed of nanoclays (MMT and C30B) dispersed in chitosan solution.

Ch solution (1.8% *w*/*v*) was obtained by dissolving Ch powder in diluted acetic acid solution (2% *w*/*v*), filtered and degassed. MMT or C30B nanoclays were dispersed in 2% (*w*/*v*) acetic acid to obtain a 0.25 wt % clay content, left for 24 h under continuous mechanical stirring and then sonicated for 1 h in a bath-type ultrasound sonicator (Elma D-78224 Singen/Htw., Singen, Germany). Clay dispersion was slowly added upon stirring to Ch solution to reach 3 wt % content of clay in nanocomposite and subsequently the mixture was homogenized for 24 h.

Composite membranes were obtained by casting film-forming solutions on the PA6 support using a casting knife (0.4 mm slit). After the casting stage, films were left in the air for the final solvent evaporation.

### 2.3. Membrane Characterization

#### 2.3.1. Fourier Transform Infrared Spectroscopy (FTIR)

Fourier transform infrared (FTIR) spectra of pure chitosan film, composite membranes and montmorillonite powder were recorded on Bruker Vertex 70 spectrometer (Bruker Optoc GmbH, Ettlingen, Germany) in ATR (Attenuated Total Reflectance) mode with diamond crystal in the range of 400–4000 cm^−1^. All spectra were recorded at the resolution of 4 cm^−1^, 16 scan passes and analyzed using OPUS 7.5 software (Bruker Optoc GmbH, Ettlingen, Germany).

#### 2.3.2. Thermogravimetric Analysis (TGA)

The differences occuring in the course of heating were evaluated by the implementation of thermogravimetric measurements. Thermogravimetric analysis (TGA) was carried out using Thermal Analysis SDT 2960 Simultaneous TGA-DTA analyzer (TA Instruments, Champaign, IL, USA) with 2–4 mg of finely cut sample pieces in an Al_2_O_3_ crucible under an air atmosphere. Experiments were run at a heating rate of 10 °C·min^−1^ in the temperature range of 20–600 °C.

#### 2.3.3. Membrane Morphology

The surface and cross-section morphologies of membranes have been observed by Scanning Electron Microscopy (SEM) using a LEO1430 VP machine (Leo Electron Microscopy Ltd., Cambridge, UK). Prior to SEM analysis, the dry samples were cut into small pieces for surface analysis or broken after immersion into liquid nitrogen for cross-section observations. Surface and cross-sections were sputtered with thin gold layer before imaging.

AFM technique was used to visualize the topological morphology and to gather information on the surface roughness. The surface topography was analyzed using a microscope with a scanning SPM probe of the NanoScope MultiMode type (Veeco Metrology, Inc., Santa Barbara, CA, USA) operating in the tapping mode, in the air, at room temperature. Membranes for AFM were prepared by cutting a piece of membrane with a size of about 1 cm × 1 cm. The roughness parameters such as the roughness average (*Ra*) and the root mean square (*Rq*) were determined for 5 µm × 5 µm scanned area (using Nanoscope v6.11 software, Bruker Optoc GmbH, Ettlingen, Germany). The roughness average (*Ra*) is an average of the absolute values of the surface height deviations measured from the mean plane:

(1) $$Ra=\frac{1}{n}\overset{n}{\underset{i=1}{\sum }}\left|y_{i}\right|$$

while the root mean square (*Rq*) is the root mean square average of height deviations taken from the mean data plane:

(2) $$Rq=\sqrt{\frac{1}{n}\overset{n}{\underset{i=1}{\sum }}y_{i}^{2}}$$

#### 2.3.4. Mechanical Properties

Tensile strength (*σ_M_*), tensile stress at break (*σ_B_*) and longitudinal modulus of elasticity (*M_t_*) were evaluated using TIRATEST 2,7025 testing machine according to the PN-EN ISO 527-1:1998 standard, using the extension rate of 100.0 mm·min^−1^.

#### 2.3.5. Contact Angle Measurements

To determine the surface hydrophilicity and wettability of all prepared membrane samples, the contact angle measurements using three various testing liquids: a non-polar diiodomethane (DIM), a bipolar glycerin (G), and a polar water (H_2_O) were performed according to the ISO 8296:2003 standard method (International Standard, 2004) by using the KRÜSS contact angle measurement system (KRÜSS Inc., Hamburg, Germany) at ca. 23 °C and 50% relative humidity. Surface free energy was determined by the Owens, Wendt, Rabel and Kaelble (OWRK) method [48]. An arithmetic mean of 10 individual measurements for each liquid was used to calculate total surface free energy (SFE, $\gamma_{S}$) and its polar ($\gamma_{S}^{p}$) and dispersive ($\gamma_{S}^{d}$) components.

#### 2.3.6. Pervaporation Experiments

**Figure 2:**
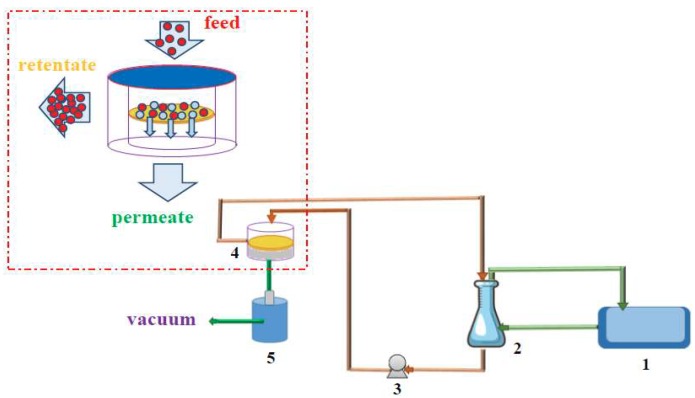
Pervaporation rig used during vacuum pervaporation experiments: (**1**) thermostat, (**2**) thermostated feed tank, (**3**) circulating pump, (**4**) membrane module, (**5**) permeate cold trap.

Pervaporation experiments were accomplished at 30 °C, using standard laboratory rig, schematically presented in Figure 2 [47]. The membrane sample was placed in a stainless steel membrane module (4) using an O-ring. The thermostated feed was circulating between module (4) and feed tank (2) applying a circulating feed pump (3) (Asti, France). Components which were preferentially transported through the membrane were collected during 1 h in one of the cold traps cooled with liquid nitrogen. Experiments were performed for membranes in contact with ethanol/water (EtOH/H_2_O) and isopropanol/water (iPrOH/H_2_O) mixtures, containing 10 and 15 wt % of water in feed with operating downstream pressure below 3 mbar.

To confirm the reproducibility of the method applied in the composite membranes preparation, two pervaporation experiments were run using two pieces of each type of membrane cut out from the different membrane sheets. Each experiment was carried out for at least 7 hours since the steady state of the system was reached.

To evaluate transport properties and separation efficiency, the permeate flux (*J*), separation factor (*β*) and Pervaporation Separation Index (PSI) were utilized. It should be pointed out that these parameters strongly depend on the experimental conditions, i.e., type of the membrane, temperature, feed composition, and pressure on the permeate side [49,50,51].

Permeate fluxes were determined gravimetrically by weighing the amount of permeate collected over a given period of time [47]. The partial flux (*J_i_*) of component *i* through the membrane was calculated from the following expression:

(3) $$J_{i}=\frac{∆m_{i}}{A\;\cdot \;∆t}$$

where Δ*m_i_* is a mass of component *i* (kg) collected over a given period of time Δ*t* (h), whereas *A* is a membrane area (m^2^).

In the case of composite membranes the fluxes were normalized to the equal thickness of 1 μm. The normalized fluxes were calculated by using Equation (4) [52]:

(4) $$J_{N,\;i}=J_{i}\cdot \;d$$

where *J_i_* is partial flux (kg·m^−2^·h^−1^), *d* is the thickness of an active layer of the membrane (μm).

The separation factor *β* allows evaluating membrane separation efficiency and was calculated by using Equation (5) [47,51]:

(5) $$\beta =\frac{y_{w}/y_{i}}{x_{w}/x_{i}}$$

where *y_w_* and *y_i_* are weight or molar fractions of water (w) and component *i* in permeate, respectively, whereas *x_w_*, *x_i_* are weight or molar fractions of water (w) and component *i* in feed.

The Pervaporation Separation Index (PSI) is a parameter allowing the comparison of the separation effectiveness of various membranes possessing different separation and transport properties [52]. PSI was calculated by using Equation (6) [47]:

(6) $${\mathrm{PSI}}_{N}=J_{N}\left(\beta -1\right)$$

where *J_N_* is total normalized flux. Total normalized flux *J_N_* is equal to the sum of partial normalized fluxes of all components present in permeate—Equation (7):

(7) $$J_{N}=\underset{i}{\sum }J_{N,\;i}$$

## 3. Results and Discussion

### 3.1. Fourier Transformed Infrared Spectroscopy

**Figure 3:**
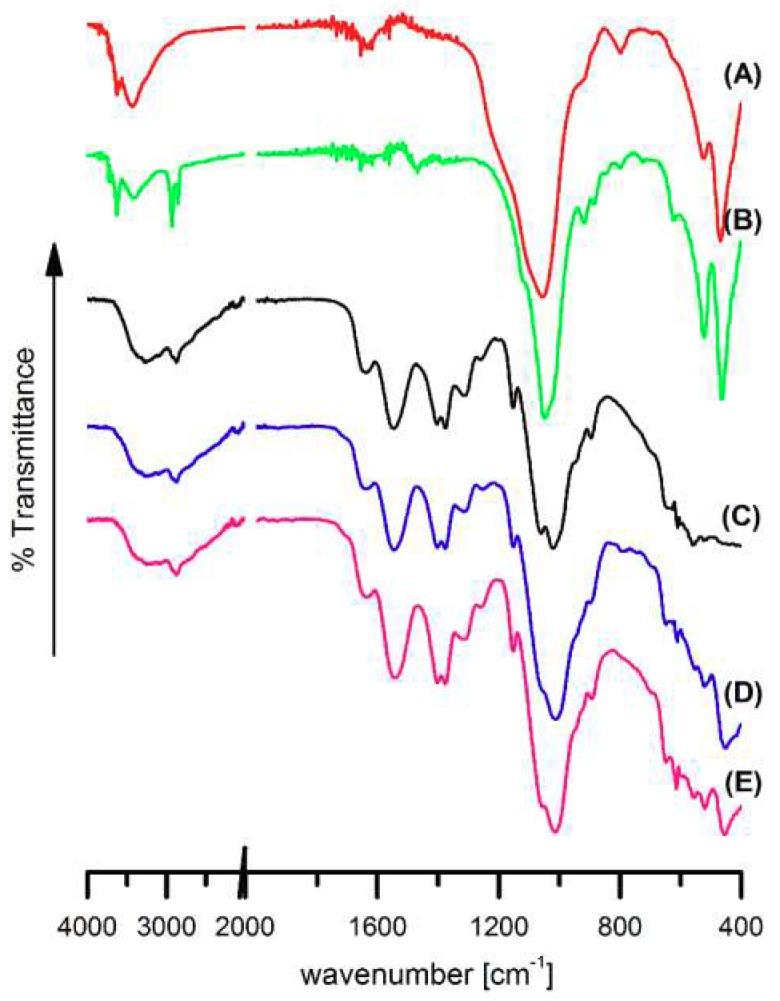
FTIR spectra of montmorillonite (**A**), Cloisite 30B (**B**) powders and PA6/Ch (**C**), PA6/Ch/MMT (**D**), and PA6/Ch/C30B (**E**) films.

FTIR spectra of nanoclay powders, neat chitosan and chitosan/clay nanocomposite films deposited on PA6 are presented in Figure 3.

In Figure 3A,B vibration band corresponding to the stretching of hydroxyl groups and cations from the octahedral sheet at 3628 cm^−1^ can be observed [53,54]. A strong band at 3438 cm^−1^ is a result of the presence of water adsorbed on the clay surface. The bands at 522 and 444 cm^−1^ refer to deformation vibrations of Si–O–Al and Al–OH, respectively [55]. In the C30B spectrum (Figure 3B) additional signals at 2926 and 2852 cm^−1^, representing stretching vibration of the C-H bonds introduced into the clay during its modification (Figure 1), are also seen [54].

In the spectrum of PA6/Ch membrane surface layer (Figure 3C) only signals characteristic for chitosan were found. Bands at 3324 cm^−1^ (O–H and N–H stretching vibrations), 1637 cm^−1^ (C=O stretching in amide group, amide I vibration), 1547 cm^−1^ (N–H bending in amide group, amide II vibration), 1152 cm^−1^ (antisymmetric stretching of the C–O–C bridge), 1060 and 1024 cm^−1^ (skeletal vibrations involving the C–O stretching) were already reported as bands characteristic for chitosan structure [56].

Some changes in the spectra of PA6/Ch after an addition of clay (Figure 3D,E) have been observed. In PA6/Ch/clay spectra, new signals characteristic of Si–O bending vibration of nanofiller used for their preparation can be seen: at 520 and 448 cm^−1^ for PA6/Ch/MMT (Figure 3D) and at 520 cm^−1^, 456 cm^−1^ for PA6/Ch/C30B films (Figure 3E). Moreover, differences in the position and in the intensity of bands in the 1250–950 cm^−1^ wavenumber range occur. This is a result of overlapping of a strong Si–O stretching band in the nanofiller and the antisymmetric stretching of the C–O–C bridge and skeletal vibrations involving the C–O stretching in chitosan.

Confirmation of the possible interaction between chitosan and clays can be found in slight shifting or intensity changing of vibrational bands. It was noticed that vibration of N-H bending in amide group in chitosan at 1547 cm^−^^1^ (Figure 3C) slightly shifts to 1551 cm^−^^1^ in PA6/Ch/MMT (Figure 3D) and 1542 cm^−^^1^ in PA6/Ch/C30B (Figure 3E). Moreover, an addition of MMT or C30B results in decreasing of the intensity of absorption band at 3324 cm^–1^, corresponding to O–H and N–H stretching vibrations. Because Si–O in-plane stretching band of clays at 1055 cm^−^^1^ (Figure 1A) and at 1047 cm^−^^1^ (Figure 1B) overlap with chitosan skeletal vibrations bands involving the C–O stretching, it was not possible to observe changes in their position in case of analyzed polymer/clay systems. These findings stay in accordance with data presented by others [53,57,58] and confirm that chitosan amino and hydroxyl functional groups forms hydrogen bonds with the silicate hydroxylated edge groups, which lead to the strong interaction between matrix and silicate layers.

FTIR analysis confirmed the presence of hydrophilic groups on the film surface characteristic for chitosan or chitosan and nanofillers.

### 3.2. Thermal Gravimetric Analysis

**Figure 4:**
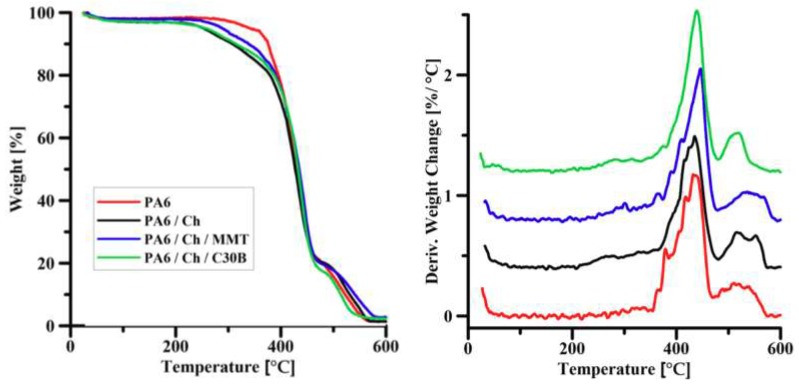
Thermograms of the pure PA6 membrane and composite membranes. Alongside: Corresponding derivatives of TGA curves for the same sample.

The thermal degradation curves of PA6 support and composite membranes (PA6/Ch, PA6/Ch/MMT, PA6/Ch/) are shown in Figure 4. TGA allows the discussion of the thermal behavior of homogenous chitosan membrane and polyamide-6 supported chitosan nanocomposite membranes filled with 3 wt % of the clay (MMT or C30B). The degradation of PA6 in air atmosphere is a two-step process (as evidenced by a small peak at ca. 543 °C), which is in a good accordance with the findings of Li et al. [59]. The thermal degradation of PA6/chitosan/clay composites under air atmosphere resembles the degradation of neat PA6. Composite membranes undergo thermal decomposition in two steps.

The degradation temperatures at 5%, 10% and 50% weight loss (*T*_5%_, *T*_10%_, and *T*_50%_) are gathered in Table 2 permitting for the comparison of membranes thermal stability. It can be seen that thermal stability of polyamide-6/chitosan/clay composites was enhanced compared to pristine chitosan film. The highest *T*_5%_, *T*_10%_ and *T*_50%_ values are noticed for polyamide-6/chitosan/clay systems. However, a weight loss of 50% occurs in practically the same temperature range for the composite membranes tested. The residual weight percentage varies from 1–3% depending on the composition of the membrane. The thermal stability increase of the nanocomposite PA6 supported membranes can be explained by the formation of a nanoscale-composite, in which the chitosan chain penetrated into the galleries of the clay [60,61]. The nanodispersion of chitosan molecules in the silicate layers not only effectively inhibited the permeation of oxygen, but also restricted their thermal motion, thus increasing the thermal stability of the prepared membrane [62,63].

An effect of nanoclay addition on the thermal stability of chitosan films stays in accordance with the results presented by Lewandowska et al. [64] for chitosan composites with MMT. The introduction of montmorillonite into the polymer matrix influences thermal properties of obtained materials. It is well known that although MMT increases the thermal stability of chitosan/MMT systems, this effect is only pronounced at low amounts of MMT, while higher MMT content causes a decrease of temperature at which degradation process starts [65]. This is the result of the formation of parallel monolayers of MMT that can form strong electrostatic interactions with chitosan. The higher loads of clay leads to the less regular structure formation, thus decreasing the strength of electrostatic interactions.

TG results show that the addition of nanofiller into chitosan matrix (Ch) deposited on PA6 support causes a shift of *T*_5%_, *T*_10%_ and *T*_50%_ temperatures toward higher values when compared to neat chitosan (Table 2).

### 3.3. Scanning Electron Microscopy

SEM surface images of PA6, neat chitosan and composite membranes (images not presented here) revealed that the surface morphology of all films is uniform, smooth and flat without any detectable pores. Thus, it can be assumed that MMT and C30B nanoparticles are highly dispersed in the Ch matrix. The strong interaction of clay with the functional group of Ch was considered as a reason for uniform particle distribution. The homogeneous morphology of obtained composite membranes may be attributed to the hydrophilic character of clay which facilities the miscibility of MMT or C30B with Ch in solution [64,66].

**Figure 5:**
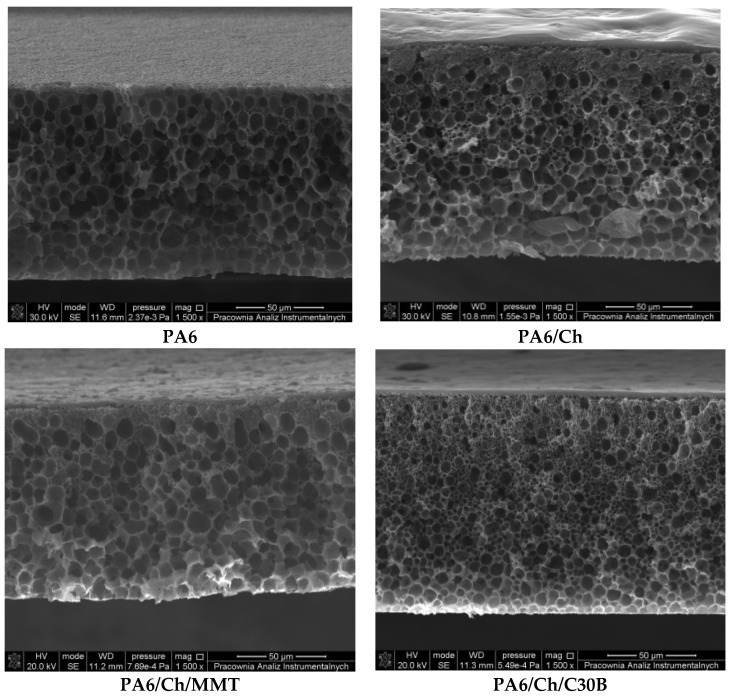
Morphology of cross-section of the porous support and composite membranes with dense selective top-layer.

Figure 5 presents the cross-sections of neat PA6 support and PA6 supported composite membranes. It can be seen that homogenous and porous support membranes were formed from pure polyamide-6 and the pore size, determined using modified bubble point method [67,68,69], was found in the range of ca. 0.150 μm. In the case of the composite PA6 membranes, the dense homogenous top layer can be seen on the porous PA6 support. SEM analysis confirmed that one side of composite membranes consists of porous PA6 structure whereas another one is smooth and homogeneous, forming dense selective membrane layer. Using an ImageJ software, it was possible to measure the thickness of the selective layer of the composite membranes. It was found that the average thickness of the selective layer was equal to 4 μm, whereas the overall membrane thickness was equal to ca. 130 μm.

### 3.4. Atomic Force Microscopy

**Figure 6:**
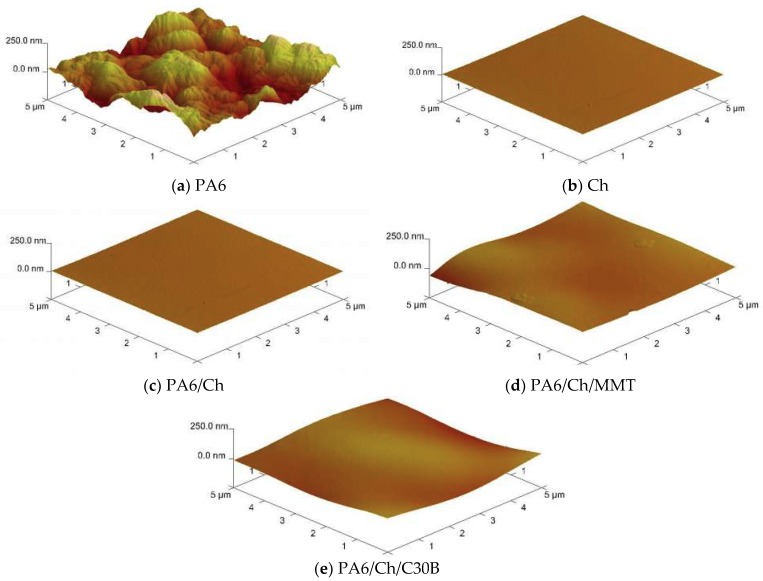
Surface morphology of PA6 (**a**), Ch (**b**), PA6/Ch (**c**), PA6/Ch/MMT (**d**), PA6/Ch/C30B (**e**).

Atomic force microscopy (AFM) was used to evaluate the surface morphology of unmodified chitosan, neat polyamide-6 and composite membranes (Figure 6). According to AFM analysis, it is possible to provide phase and amplitude images to support additionally the height-profile images for a better insight into the membrane topography. The corresponding roughness values such as Ra and Rq are gathered in Table 3. The images depict differences between the surface of pure chitosan (Ch), homogeneous polyamide-6 and chitosan-based composite membranes (PA6/Ch/MMT, PA6/Ch/C30B). From Figure 6, as well as from the data presented in Table 3 it can be concluded that the PA6 support possesses the highest surface roughness (*Ra* = 63.1 nm, *Rq* = 50.3 nm). This stays in a good agreement with the porous structure of PA6 revealed by SEM analysis (Figure 5). The non-uniform PA6 surface structure is a result of pores that can be seen in PA6 cross-section (Figure 5). The composite membranes (Table 3, Figure 6) exhibit a more uniform and smoother surface with lower roughness parameters being only slightly higher than those measured for neat Ch surface. Furthermore, AFM images show differences in surface properties of PA6/Ch/MMT films and PA6/Ch/C30B film. The results indicate that the introduction of nanofiller significantly influences the surface properties of prepared membranes. The surface of chitosan films has been altered by the addition of MMT and C30B. The increase of surface roughness of polyamide-6/chitosan/MMT and polyamide-6/chitosan/C30B in comparison to polyamide-6/chitosan film is probably due to the interactions between biopolymer and inorganic compounds [70].

### 3.5. Contact Angle Measurements

Contact angle measurements were used for the evaluation of effectiveness of chitosan and chitosan/nanoclay layer deposition on PA6 support. Moreover, based on the obtained results, it was possible to compare an effect of nanoclay addition on the membrane surface hydrophilicity.

Total surface free energies (SFE) as well as dispersive and polar components of neat PA6 support, neat chitosan film and PA6 supported Ch and Ch/clay films are summarized in Table 4. AFM analysis revealed that the surface roughness of all specimens is less than 50 nm or close to 50 nm (PA6) (Table 3). Thus, contact angle and SFE values are not influenced by the surface topography [71,72]. As can be seen, there is a high contrast in contact angles and SFE values between PA6 and Ch. It is well known that PA6, due to the presence of amide (-CONH) functional groups, possesses slightly hydrophilic properties characterized by SFE = 46.5 mN·m^−1^ [73,74] which is in good accordance with the data presented in Table 4: SFE = 47.61 mN·m^−1^. The surfaces of neat chitosan film, due to the large number of hydrophilic –OH and –NH_2_ groups, can be regarded as a hydrophilic one with θ_DIM_ significantly higher than that of PA6.

The values of contact angle of diiodomethane on composite PA6 supported membranes are similar to those on neat chitosan ones and allow to confirm successful deposition of pristine chitosan as well as chitosan/clay solutions on PA6 support. Moreover, low standard deviations values of measured contact angles proved the uniform distribution of the deposited layer.

It can be noticed that there are only small changes in hydrophilic-hydrophobic balance in the chitosan layer after the introduction of nanoclay. The lowest contact angle of diiodomethane on PA6/Ch/C30B film surface suggests that this is the most hydrophobic surface, while PA6/Ch/MMT the most hydrophilic one. This is due to the highly hydrophilic nature of MMT clay and less hydrophilic characteristic of C30B [75], being a MMT derivative obtained by using an organic quaternary ammonium salt (Figure 1).

The *γ_S_* for Ch is low and corresponds to the data presented in literature [76]. After the addition of either MMT or C30B the films, the total SFE value changes are mostly related to the decrease or increase in the polar $\gamma_{S}^{p}$ component value, whereas the dispersive $\gamma_{S}^{d}$ component changed only a little upon the clay addition.

### 3.6. Mechanical Properties

Chitosan exhibits good film forming properties. The solvent evaporation technique is the most often used for Ch membranes formation, resulting in uniform, non-porous dense structures [77].

**Figure 7:**
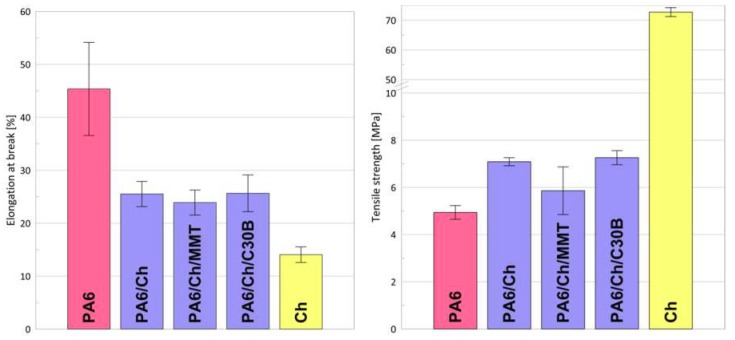
Elongation at break and tensile strength of PA6 support, pristine chitosan and composite chitosan membranes.

The values of the elongation at break (*ε_B_*) and tensile strength (*σ_y_*) are presented in Figure 7. As it can be seen, the pristine chitosan membrane is characterized by a low mechanical resistance and brittle character, possessing *ε_B_* equal to 14%. Bearing in mind that Ch films swell in contact with external fluids, using a pristine chitosan membrane in separation processes requires higher film thickness. Higher membrane thickness leads to lower permeate flux. To overcome that problem, composite membranes were obtained with thin chitosan surface, selective layer and PA6 as a support.

Because of polyamide versatility, this polymer is one of the most widely used engineering thermoplastics being commonly used as a membrane forming material for microfiltration, nanofiltration, ultrafiltration, and reverse osmosis [78]. Polyamide-6 has very good film forming properties. As can be seen in Figure 7, application of a phase inversion method for the preparation of PA6 films results in a material of good mechanical and better elastic properties: almost three times higher elongation at break than for pristine chitosan. Casting of a thin chitosan layer onto PA6 support caused formation of composite films with higher elasticity than for the Ch alone. 

To improve the mechanical stability of different polymers, the nanocomposite approach is often applied [79]. Data presented in Figure 7 indicate that there are only small differences between PA6/Ch and PA6/Ch/MMT, PA6/Ch/C30B, regarding the mechanical properties. As it was already suggested in the literature [80], an addition of organoclays should improve the mechanical properties of different bio-polymer based films if the amount of nanoclay does not exceed 5 wt %. However, Araújo et al. [81] found that mechanical properties of polyethylene (PE) based nanocomposites are close to those of the pure PE. Xu [61] showed that tensile strength of Ch/MMT films increases for 1 and 3 wt % of MMT and then decreases for 5 wt % of nanofiller while all *ε_B_* of Ch/MMT are lower than for the Ch one. On the contrary, the addition of Cloisite 30B to the chitosan solution does not affect *σ_y_* value but significantly decreases *ε_B_*. Changes in mechanical properties of chitosan nanocomposites are closely related to the internal structure of nanocomposite, and in case of chitosan, also to the polymer properties (molecular weight, degree of deacetylation, crystallinity) and synthesis procedure (a type of solvent, temperature, film forming method) [82,83,84]. Because of numerous factors affecting mechanical properties of chitosan nanocomposites and taking into account that PA6 supported films were analyzed, our results cannot be simply compared with the data of other researchers. Based on the data presented within this research, it can be assumed that the addition of nanoclay does not improve the mechanical properties of composite films and that *σ_y_*, *ε_B_* values are mostly related to the applied PA6 support. To this extent, our results are similar to the results presented elsewhere [61,85].

### 3.7. Pervaporation Properties of Membranes

The pervaporation efficiencies of homogenous and composite PA6 based membranes in contact with alcohol/water (90/10 wt % and 85/15 wt %) mixture are gathered in Table 5. Binary water/ethanol and water/isopropanol solutions of different composition were used as feed. The thicknesses of prepared membranes were different, therefore the fluxes were normalized to the uniform membrane thickness of 1 μm, according to Equation (4). Data gathered in Table 5 are mean values of all obtained results (i.e., for the first and second piece of the membrane). Small values of standard deviation values confirm additionally the stability of separation efficiency during the pervaporation process.

Data presented in Table 5 show that homogenous chitosan membrane, as well as composite membranes, selectively transported water from its binary mixture with ethanol or isopropanol. It was found that the best separation factor was obtained for the PA6/Ch/C30B membrane. This preferential transport of water is a result of the hydrophilic nature of chitosan and nanocomposite films based on chitosan as it was proven by the contact angle measurements. As it can be seen (Table 5), the composite membranes, when compared to one-layer neat chitosan one, exhibit two orders of magnitude higher total flux. The PA6/Ch/C30B and PA6/Ch/MMT membranes give a higher flux than PA6/Ch membrane and moreover, PA6/Ch/C30B produces the highest normalized flux among all studied membranes. Based on the results presented in Table 5 it can be seen that the addition of clay into chitosan selective layer deposited on PA6 support plays a beneficial role on separation parameters of these membranes when compared to non-filled supported PA6/Ch membrane. PA6/Ch/C30B was found to be the most effective membrane towards water recovery, regardless of the water–alcohol mixture used in this study.

**Figure 8:**
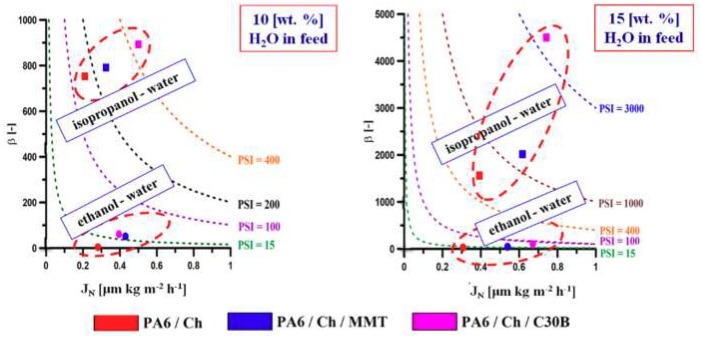
Comparison of various membranes’ performance during recovery of water from its binary alcohol mixtures.

Pervaporation Separation Index (PSI)—Equation (6) was utilized for the assessment of the membranes performance in a pervaporation process. The higher the PSI value, the more efficient is the membrane used in PV. As it can be seen from the data presented in Figure 8, separation using PA6/Ch/C30B membrane leads to the highest efficiency for all binary mixtures used in this work. Since chitosan is relatively hydrophilic in a sense that it has a polar amino group attached to a repeat unit of the main chain, it is understandable that membranes based on chitosan show high water separation efficiency [28]. When considering different water-alcohol mixtures of the same water content, higher total fluxes were obtained for the water-isopropanol mixture than for the water-ethanol one. Stronger affinity of chitosan to water and the fact that molecular size of water is smaller than that of isopropanol would make the chitosan membrane more selective to water. The total fluxes decrease with increasing alcohol concentration in the feed solution. Owing to the high hydrophilicity of the chitosan material, the chitosan membranes swell more significantly in the solution of higher water content. As the water concentration in feed increases, the amorphous regions of the membrane are more swollen, and the polymer chains become more flexible and increase the space available for diffusion, thus decreasing the energy required for diffusive transport through the membrane. Finally, the total fluxes through the highly hydrophilic chitosan membranes increase with an increase in water concentration in the feed solution. The presence of clay in a polymer matrix and the use of a porous support could greatly enhance the separation factor of Ch membrane.

Comparison of presented results with data given in literature for different chitosan-based membranes can be difficult due to the fact that many factors affect the transport properties through the biopolimeric membranes in PV, e.g., temperature of the feed solution, difference in vapor pressure and thus in driving force, different membrane structure and thickness etc. Molecular and supermolecular structure of chitosan, a copolymer with different degrees of deacetylation and various molecular weight, also play an important role. A large extent of chitosan sources and applied PV operating conditions makes a comparison of fluxes and separation factors complicated, especially if non-normalized fluxes are given.

As we have stated in the introduction, even if several chitosan-based natural mineral clay nanocomposite films were obtained, only Choudhari et al. [34] evaluated their application in the PV process. Chitosan membranes modified with Na^+^-MMT clay (0, 5, 10, and 15 wt %) were used in the separation of water–iPrOH mixtures (5–25 wt % of water in the feed) at 30, 40, and 50 °C. Authors reported that an increase of Na^+^-MMT clay content in the quaternized chitosan matrix decreases total permeation flux but simultaneously increases separation efficiency. While assessing the membranes’ efficiency, it was clearly noted that both total flux and flux of water are overlapping each other, signifying that the composite membranes developed by the authors are highly selective toward water, also proved in present research. The Pervaporation Separation Index values confirmed that the clay-incorporated membranes except membrane containing 15 wt % of clay demonstrated an excellent PV performance. Among the membranes, the membrane containing 10 wt % of Na^+^-MMT clay exhibited the highest separation selectivity of 14992 and with a total permeation flux of 14.23 × 10^−2^ kg·m^−2^·h at 30 °C for 10 wt % of water in the feed. Based on the membrane thickness given by the authors (*d* = 40 μm [34]) we have found normalized flux equal to 5.7 μm·kg·m^−2^·h being ca. one order of magnitude higher than this we noticed for PA6/Ch/C30B membrane. This can be explained by high clay content. Choudhari et al. [34] also found that an increase in temperature resulted in an increase of permeation rate while suppressing the selectivity. Authors supposed that this phenomenon was attributed to higher vapor pressure and lower interaction between permeants and membrane at higher temperatures. Even if Choudhari et al. [34] obtained promising results, they do not evaluate mechanical properties of MMT-filled membranes. 

Other researchers [28] compared the separation properties of homogeneous and composite chitosan-based membrane. Polysulfone (PSf) was used as a porous support. It was found that in the PV separation of water–iPrOH mixtures (5–90 wt % of water in the feed) at 30, 40, 50 and 60 °C, only water was transported across both investigated membranes. This was explained by a smaller molecular size of water compared to that of isopropanol. The authors also noticed that along with the increase of the isopropanol concentration in the feed, the flux of water decreased. In the case of the chitosan membrane, the maximum isopropanol flux was obtained at the 25 wt % alcohol concentration whereas in the case of the chitosan/polysulfone membrane, it was obtained at 40 wt % of isopropanol. This was explained by the influence of water on alcohol transportation. The decrease of water content in the feed increases friction between water and alcohol molecules in the membrane. Ghazali et al. [28] also proved that total permeate flux as well as water flux through PSf supported membranes is higher compared to the homogeneous one. These findings remain in agreement with the data presented in Table 5. On the contrary to the data in Table 5, Ghazali et al. [28] registered that chitosan membranes deposited on porous support exhibited lower separation factor than non-supported chitosan membrane.

Ge et al. [86] applied the solvent evaporation method to obtain H_2_SO_4_ crosslinked chitosan membranes and put attention on the correlation between different preparation conditions and the PV performance of membranes. Different drying conditions and various concentrations of crosslinking solution were used. Authors discussed the influence of chosen parameters (chitosan concentration in casting solution, temperature and time of drying) on the properties of the prepared membranes. Water-ethanol mixtures (10, 30 and 50 *v*/*v* of water in the feed) were separated through the membranes at 30, 40, 50, and 70 °C. The results showed that the heating temperature had the greatest influence on the membrane transport properties. The membranes separated 90/10 wt % ethanol/water mixture most effectively at 70 °C, which was explained by a high degree of crystallinity of membranes at that temperature. The influence of the degree of chitosan crosslinking on the azeotrope separation efficiency was also discussed. The increase of the crosslinking degree of chitosan membranes led to the increase of selectivity and the decrease of the permeate flux.

## 4. Conclusions

In this work, chitosan composite membranes supported on polyamide-6 were prepared and evaluated in the pervaporation separation of alcohol/water mixtures. FTIR analysis confirmed the strong interaction between Ch and clays and possible intercalation of Ch chain between the clay sheets. SEM images confirmed the successful deposition of thin, dense, homogenous selective chitosan and chitosan/nanoclay layers onto the porous PA6 support. PA6 supported composite membranes exhibited improved mechanical properties in comparison to neat non-supported chitosan membrane. Moreover, an addition of nanoclays into chitosan matrix affected the morphology yet insignificantly improved the thermal properties of the resulting membranes. According to AMF images, composite membranes filled with nanofillers show much higher roughness than the PA6/Ch membrane. 

All chitosan-based membranes preferentially transport more polar component (water) of the water-alcohol mixtures with permeate fluxes noticeable higher when thin chitosan film was used as a separation layer. Increasing of flux through the PA6 supported membranes was accompanied by higher pervaporation separation index (PSI). The highest PSI value was observed for Ch/PA6 films modified with Cloisite 30B nanoclay. Thus, it can be concluded that both chitosan deposition on porous support as well as nanoclay addition into selective chitosan layer beneficially affected separation performance of chitosan membranes in PV.

## Figures and Tables

**Table 1:** Chemical composition of montmorillonite.

**Compound**	SiO_2_	Al_2_O_3_	Fe_2_O_3_	CaO	MgO	Na_2_O	K_2_O
**Content (wt %)**	73.0	14.0	2.7	0.2	1.1	0.6	1.9

**Table 2:** TGA data for all analyzed samples.

Sample	Temperature/°C at mass loss			Residual mass at 600 °C
	*T*_5%_	*T*_10%_	*T*_50%_	(%)
MMT	86.1	592.3	-	90.4
C30B	244.6	288.1	-	77.5
Ch	66.9	124.4	332.0	0.9
PA6	341.1	375.7	431.1	1.3
PA6/Ch	257.3	307.6	427.3	1.4
PA6/Ch/MMT	288.3	338.0	434.9	2.7
PA6/Ch/C30B	297.9	314.2	432.8	2.3

**Table 3:** The roughness parameters (*Ra* and *Rq*) of prepared membranes.

Membrane	*Ra* (nm)	*Rq* (nm)
PA6	63.1 ± 0.5	50.3 ± 1.1
Ch	1.9 ± 0.3	1.6 ± 0.2
PA6/Ch	7.2 ± 0.3	5.3 ± 0.5
PA6/Ch/MMT	13.1 ± 0.1	10.8 ± 0.5
PA6/Ch/C30B	16.2 ± 0.7	12.6 ± 0.8

*Ra*—the roughness average, *Rq*—the root mean square.

**Table 4:** Contact angle (°) and surface free energy values for PA6 support and composite chitosan membranes.

Sample	Contact angle (°)			Surface free energy (mN·m^−1^)		
	H_2_O	DIM	G	$\mathrm{total}\;\gamma_{S}^{}$	$\mathrm{dispersive}\;\gamma_{S}^{d}$	$\mathrm{polar}\;\gamma_{S}^{p}$
PA6	51.5 ± 2.1	20.6 ± 0.8	57.5 ± 1.4	47.61	45.31	2.30
Ch	77.3 ± 3.6	47.6 ± 1.9	71.0 ± 2.1	35.6	33.97	1.63
PA6/Ch	74.9 ± 3.3	47.2 ± 1.6	64.7 ± 1.8	36.53	32.14	4.39
PA6/Ch/MMT	74.9 ± 2.3	47.6 ± 3.2	65.8 ± 4.1	36.13	32.21	3.92
PA6/Ch/C30B	68.9 ± 1.2	46.1 ± 1.5	60.7 ± 4.6	38.04	31.65	6.40

Where DIM—diiodomethane, G—glycerin.

**Table 5:** Efficiency of homogenous and composite chitosan membranes in the separation of water-alcohol mixtures.

Membrane	Thickness (μm)	EtOH/H_2_O (90/10 wt %)		EtOH/H_2_O (85/15 wt %)		iPrOH/H_2_O (90/10 wt %)		iPrOH/H_2_O (85/15 wt %)	
		*β* (-)	*J_N_* (μm·kg·m^−2^·h^−1^)	*β* (-)	*J_N_* (μm·kg·m^−2^·h^−1^)	*β* (-)	*J_N_* (μm·kg·m^−2^·h^−1^)	*β* (-)	*J_N_* (μm·kg·m^−2^·h^−1^)
**Ch**	36.5 ± 0.1	18 ± 2	0.0016 ± 0.0001	15 ± 2	0.0035 ± 0.0002	431.0 ± 0.5	0.0073 ± 0.0003	980.0 ± 0.6	0.0057 ± 0.0011
**PA6/Ch**	124.3 ± 0.1	39 ± 1	0.281 ± 0.005	31 ± 3	0.307 ± 0.001	756.0 ± 1.0	0.210 ± 0.002	1562.0 ± 0.5	0.392 ± 0.006
**PA6/Ch/MMT**	110.8 ± 0.3	51 ± 1	0.430 ± 0.003	45 ± 1	0.539 ± 0.005	791.0 ± 0.9	0.325 ± 0.002	2017.0 ± 0.7	0.617 ± 0.008
**PA6/Ch/C30B**	130.1 ± 0.1	60 ± 2	0.395 ± 0.003	105 ± 1	0.671 ± 0.004	893.0 ± 0.8	0.501 ± 0.003	4500.0 ± 1.2	0.742 ± 0.002
